# Methods Used for Endotracheal Tube Cuff Inflation and Pressure Verification in Veterinary Medicine: A Questionnaire on Current Practice

**DOI:** 10.3390/ani12223076

**Published:** 2022-11-08

**Authors:** Iris Veen, Janny C. de Grauw

**Affiliations:** Department of Clinical Sciences, Faculty of Veterinary Medicine, Utrecht University, 3584 CM Utrecht, The Netherlands

**Keywords:** cuff pressure, endotracheal tube cuff, cuff pressure measurement, cuff inflation

## Abstract

**Simple Summary:**

Endotracheal intubation is the process whereby a tube is placed in the trachea during anaesthesia to assist breathing, administer anaesthetic gases and prevent fluids from entering the trachea. Endotracheal intubation is a routine procedure in veterinary anaesthesia, yet no guidelines exist for establishment of a sealed airway. Through an online survey of veterinary professionals who administer anaesthesia, we aimed to assess specific aspects of current practice of endotracheal intubation in veterinary medicine. The pressure in the cuff (a balloon at the end of the tube that, when inflated, contacts the trachea to seal off the airway) was measured by almost one-third of respondents in cats and dogs but by less than one-tenth of respondents in farm animals and horses. Respondents seemed to target a similar cuff inflation pressure, regardless of species, although a higher pressure was more often selected in horses compared to dogs, cats and farm animals. The preferred technique to verify cuff seal was the same in dogs, cats and farm animals, whereas in horses, a different technique was preferred. Cuff pressure measurement remains uncommon in veterinary anaesthesia. The development of recommendations for cuff inflation, including cuff pressure ranges for various species, can help to improve practice.

**Abstract:**

Endotracheal intubation is a routine procedure in veterinary anaesthesia, yet no consensus guidelines exist for endotracheal tube (ETT) cuff inflation and pressure measurement. The aim of this study was to assess current practice of ETT cuff inflation and seal verification in veterinary medicine. An online questionnaire was distributed among veterinary professionals who administer anaesthesia, comprising six demographic and twelve ETT cuff-related questions per species. N = 348 questionnaires were completed. Cuff pressure was measured by 30% of respondents in cats, 32% in dogs and 9% in both farm animals and horses. Anaesthesia diplomates were not more likely to measure cuff pressure than others, except in cats (OR: 1.8; 95% CI: 1.1–2.9). The most frequently selected recommended range of cuff pressure was 20–30 cm H_2_O, regardless of species, although >30 cm H_2_O was selected significantly more often in horses compared to dogs, cats and farm animals. The preferred technique to verify cuff seal was minimal occlusive volume in dogs, cats and farm animals, whereas in horses, the preferred method was verification of normal capnogram waveform. ETT cuff pressure measurement remains uncommon in veterinary anaesthesia. The development of consensus recommendations for cuff inflation, including evidence-based target cuff pressure ranges for various species and different ETT models or materials, can help to improve practice.

## 1. Introduction

Endotracheal intubation is routinely performed in veterinary medicine; however, no consensus guidelines exist for endotracheal tube (ETT) cuff inflation and optimal target range for cuff pressure. There may also be relevant differences between species and/or between ETT make, model or material.

In dogs, digital palpation of the pilot balloon and inflation to minimum (audible) occlusive volume proved ineffective to ensure inflation of the ETT cuff within the optimal pressure range [1]. In a similar study in cats, pilot balloon palpation, minimum occlusive volume and loss of resistance techniques all performed poorly at achieving predetermined ETT cuff pressures [2]. To the best of our knowledge, there is no available literature on cuff inflation techniques and their relative performance in horses and farm animals.

The recommended ETT cuff pressure range of 20–30 cm H_2_O most commonly cited in the medical literature is based on decreases in tracheal capillary perfusion when ETT cuff pressures over 30 cm H_2_O were used in rabbits [3] and humans [4]. However, this recommendation pertains to high-volume–low-pressure cuff on polyvinyl chloride (PVC) ETTs, which are frequently used for endotracheal intubation in humans and companion animals. In large animals, silicone ETTs fitted with low-volume–high-pressure cuffs are regularly used. These cuffs are elastic and known to have greater compliance than PVC cuffs [1,5]. As a result, no simple linear relation between intracuff pressure and tracheal transmural pressure exists for these tubes [6,7,8]. In horses, an ETT cuff pressure of more than 80 cm H_2_O was required to provide a seal sufficient to prevent liquid leakage around the low-volume–high-pressure cuff silicone 30 mm ETT [9], which is much higher than the 20–30 cm H_2_O reported for PVC ETTs.

As there are no veterinary guidelines and literature data are contradictory or incomplete, in this study, we sought to assess current practice for ETT cuff inflation and pressure verification in veterinary anaesthesia.

The first aims of the present study were to evaluate (I) common ETT material properties and reuse of ETTs in various species, (II) techniques used for ETT cuff inflation and cuff pressure measurement, (III) the target range of cuff pressure used, (IV) preferred techniques to establish ETT cuff seal and (V) the frequency and timing of rechecking ETT cuff seals. A secondary aim was to investigate the influence of training level (diplomate vs. first-opinion veterinarian) and years of experience on cuff inflation practice.

## 2. Materials and Methods

### 2.1. Questionnaire

An Internet-based questionnaire cross-sectional study was conducted via Facebook and through e-mail list servers. The questionnaire (Appendix A) was developed based on recent literature [10], expert opinion and the checklist for reporting results of Internet e-surveys [11]. Deviation from CHERRIES guidelines is mentioned were applicable. The questionnaire was piloted for usability and technical functionality among Utrecht University senior veterinary anaesthesia providers.

The final survey included six demographic questions about gender, country of work, job title, place of work (first-opinion practice, referral practice, university teaching hospital or other), years of veterinary experience and animal species routinely intubated (dogs, cats, farm animals and/or horses). Eight additional questions per species category covered the material of ETTs and reuse of ETTs, techniques used for ETT cuff inflation, preferred techniques used to establish an adequate tracheal seal and, if the ETT cuff seal is routinely rechecked, what the participant thinks the recommended range of cuff pressure is; finally, the participants were asked to prioritise items associated with ETT cuffing.

Adaptive questioning was used in two specific questions. If participants indicated a cuff pressure measurement device was used for ETT cuff inflation, follow-up questions asked which cuff pressure measurement device was used and what cuff pressure was targeted. If participants answered that the ETT cuff was rechecked, two further questions asked with respect to when and how. Therefore, in addition to the six demographic questions, respondents were asked eight to twelve questions per species category, depending on their answers.

Institutional ethical approval for this study was sought but ruled not to be required under Dutch legislation (documentation on file).

### 2.2. Distribution of the Questionnaire and Informed Consent

Study participants were recruited via a closed Facebook community for Dutch veterinarians (“Het Dierenartsengilde”), which is a private community for veterinarians only. Furthermore, study participants were recruited through the e-mail list servers of the European College of Veterinary Anaesthesia and Analgesia (ECVAA and the ACVA-L server.

The questionnaire was drafted and provided to all study participants in English. The survey remained open from the 9th of December to the 31st of December 2021 (22 days). An e-mail reminder to participate was sent 2 weeks after the opening invitation of the survey. The questionnaire was not password-protected and was developed and made available using Qualtrics XM online survey software.

Informed consent was obtained before the questionnaire could be started. Study participants were informed about the purpose of the study, that that data collection was anonymous and that they could opt out at any moment. Participation in the questionnaire was voluntary, and no incentives were offered for completion of the survey.

### 2.3. Target Population

The target population consisted of veterinarians (first-opinion practitioners, as well as residency-trained anaesthesiologists, including diplomates of the ECVAA and ACVAA) and veterinary anaesthesiology nurses/technicians who routinely perform endotracheal intubation for anaesthesia. Study participants who indicated that they do not regularly perform endotracheal intubation in dogs, cats, farm animals and/or horses were immediately redirected to the end of the survey.

The closed Facebook community for Dutch veterinarians (“Het Dierenartsengilde”) has a membership count of 3500 individual veterinarians, and the ECVAA and ACVA-L e-mail list servers comprise of an estimated 415 and 1000 members, respectively.

### 2.4. Data Handling

Survey completeness was checked for every respondent, and incomplete questionnaires were excluded from analysis. Software (IBM SPSS 27) was used to search for duplicate entries; none were detected. The final question on prioritising items associated with ETT cuffing (see Appendix A) was excluded from analysis post hoc, as participant feedback indicated it was not sufficiently intuitive how respondents could drag and drop the items to rank them from highest to lowest importance; therefore, when the original order of items appeared as the answer, we could not be sure whether or not this was intentional.

### 2.5. Statistical Analysis

Descriptive analysis was performed on answers to the demographic questions. The Shapiro–Wilk test was used to assess normality. A Wilcoxon signed-rank test was performed for comparison of differences between species for the same questionnaire item. Odds ratios were calculated to compare the odds for dichotomized respondent demographic categories diplomate/non-diplomate, first-opinion practice/non-first-opinion practice and >10 years of experience/≤10 years of experience. All statistical analyses were performed in IBM SPSS 27.

## 3. Results

A total of 425 people participated in the study, with 348 questionnaires fully completed and included for analysis. Owing to the complete anonymization of the questionnaire, it was not possible to calculate a view rate (ratio of unique survey visitors/unique site visitors). The participation rate (ratio of first survey page visitors/visitors who agreed to participate) and completion rate (ratio of users who finished the survey/users who agreed to participate) were 407/423 (96%) and 348/423 (82%), respectively [11]. Results and statistics are presented in Table 1 and Table 2. A comprehensive overview of all odds ratios for respondent answers can be found in Appendix B.

### 3.1. Demographics

Comprehensive demographic details of the study participants can be found in Appendix C. Almost half of the study participants were first-opinion veterinarians, with 95% practicing in the Netherlands; only 2% of respondents were anaesthesiology nurses/technicians. Nearly 10% of the study participants were anaesthesia residents, whereas ACVAA and ECVAA diplomates comprised of 14 and 18% of respondents, respectively. Respondents worked in first-opinion practice (43%), referral practice (22%) and university teaching hospitals (32%).

Study participants indicated regularly performing endotracheal intubation in dogs (97% of participants), cats (93%), farm animals (34%) and horses (37%). Almost one-third (32%) of the study participants routinely performed endotracheal intubation in all four species categories. Diplomates were overrepresented in the group that intubated farm animals and horses (52% and 54%) compared to those intubating small animals (31%). Intubation of farm animals and horses was rarely performed by respondents working in first-opinion practice (2% in both).

### 3.2. Material and Reuse of Endotracheal Tubes

Silicone was the most commonly used ETT material in dogs, farm animals and horses, whereas in cats, PVC was the most frequently selected material for ETT. Overall, silicone was far more common as an ETT material type in farm animals and horses (91% and 86%) compared to dogs and cats (67% and 45%; see also Table 1).

In line with this result, reuse of ETTs in dogs and cats was significantly less common than in horses and farm animals. Working in first-opinion practice neither increased nor decreased the odds of re-using the ETT (Table 2).

### 3.3. Methods Used for Cuff Inflation, Cuff Pressure Measurement and Target Range of Cuff Pressure Used

The most commonly selected preferred method for cuff inflation across species was a syringe filled with air (Table 1). Respondent answers indicated cuff pressure is measured in 30% of dogs, 32% of cats and 9% of farm animals and horses. Anaesthesia diplomates were not more likely to measure cuff pressure than others, except in cats (see also Table 2). Working in first-opinion practice did not affect the likelihood of measuring cuff pressure in dogs and cats (see also Table 2), whereas more than 10 years of work experience increased the likelihood of measuring cuff pressure in cats (see also Table 2). The most frequently used cuff pressure measurement device was an AG Cuffill^®^ in cats and a handheld sphygmomanometer in the three other species groups. Among study participants indicating the use of a cuff pressure measurement device, 34% did not enter what target cuff pressure was used. After exclusion of these responses, the most commonly targeted cuff pressure range when using a cuff pressure measurement device was 20–30 cm H_2_O in cats, dogs and farm animals. In horses, however, the most frequently selected targeted cuff pressure range was >30 cm H_2_O. Although there was no significant difference in cuff pressure used between species, respondent answers with respect to their perception of the recommended range of cuff pressure were differed significantly between species.

### 3.4. Preferred Techniques Used to Establish Endotracheal Tube Cuff Seal

The preferred technique to verify cuff seal was minimal occlusive volume in dogs, cats and farm animals, whereas in horses, the preferred technique was verification of normal capnogram waveform (Table 1). Diplomates were more likely to use ≥3 different techniques to verify cuff seal than other respondents (Table 2).

Preference for a technique was largely dictated by training, as half of all respondents selected “I was taught to do it this way” when asked to explain their preference.

### 3.5. Frequency and Timing of Rechecking Endotracheal Tube Cuff Seal

The most frequently selected answer option for rechecking cuff seal was “Yes, sometimes” for all species (Table 1). Whereas the frequency of never rechecking cuff seal was not dependent on species, the odds of never rechecking the cuff were higher in first-opinion practice in dogs and cats and lower in cats for respondents who had more than 10 years of work experience (Table 2). Interestingly, no diplomate selected the option of never rechecking the cuff in dogs, and in cats, the odds of diplomates never rechecking cuff seal were significantly lower compared to non-diplomates (Table 2).

Rechecking of ETT cuff seal was most frequently performed only upon specific indication, with the most frequently selected specific indication being suspicion of cuff leakage.

The preferred technique for rechecking cuff seal was similar to the initial method used to establish the cuff seal; minimal occlusive volume was the preferred technique in dogs, cats and horses (57, 94 and 53% of answers, respectively) and verification of normal capnogram waveform in horses (56%).

## 4. Discussion

It is remarkable that no evidence-based consensus guidelines exist in veterinary medicine for such a common yet crucial procedure as creation of a tracheal seal after endotracheal intubation of various species. The American Animal Hospital Association Anaesthesia and Monitoring guidelines only state that, “A properly inflated cuff on a conventional ETT is necessary to create a seal for adequate positive pressure ventilation and avoid inhalant leakage, being aware that over-inflation may cause tracheal damage” [12]. The Association of Veterinary Anaesthetists recommended requirements for airway management when performing general anaesthesia of dogs, cats and horses merely stipulate that one must “ensure the animal’s airway is patent” [13]. Cuff pressure measurement has been recommended in human [8,14,15,16] and veterinary medicine [1,9] to guide ETT cuff inflation to sufficient yet not excessive pressure. Importantly, whereas under-inflation may give rise to inhalant leakage and aspiration, cuff over-inflation can lead to tracheal mucosal damage [9,17,18,19], tracheal necrosis [20]), tracheal perforation [21], airway obstruction [22] and postoperative complications, such as sore throat [23,24].

Despite these risks, in this survey, cuff pressure was measured by less than one-third of respondents in dogs and cats and by less than one in ten in farm animals and horses. Advanced anaesthesia training and more years of work experience did not seem to considerably affect the practice of cuff pressure measurement, as diplomates and respondents with more than 10 years of work experience were more likely to measure cuff pressure only in cats, and working in first-opinion practice in general did not influence the likelihood of measuring cuff pressure. One might expect that individuals with advanced anaesthesia training would be generally more aware of the literature on the risks of ETT cuff under- and over-inflation.

The infrequent use of ETT cuff pressure measurement in veterinary specialist or first-opinion practice, as apparent from this survey, may, in part, also be due to the absence of evidence-based guidelines for recommended cuff pressure ranges. The often generically cited recommended ETT cuff pressure range of 20–30 cm H_2_O is derived from studies that showed impaired tracheal capillary perfusion when pressures over 30 cm H_2_O were used in rabbits [3] and people [4]. However, venous and lymphatic drainage of the trachea is already impaired at much lower pressures [3], and hypotension can also negatively impact tracheal blood flow [25]. Moreover, the relation between ETT cuff pressure and tracheal transmural pressure is highly dependent on make, material and ETT diameter [1,5] and therefore cannot simply be extrapolated from one species to another. The silicone ETTs that are typically used in large animal anaesthesia feature low-volume–high pressure cuffs, which are more compliant than polyvinyl-chloride (PVC) cuffs [1,5]; therefore, a different cuff pressure target range seems to be necessary to provide an adequate seal in silicone compared to similarly sized PVC ETTs [1,9].

In a human anaesthesia survey, variations in cuff pressure targets were noted, with 11% of respondents targeting 10–20 cm H_2_O, 38% 21–25 cm H_2_O and 50% 26–30 cm H_2_O [26]. In the current study, the range of cuff pressure that was targeted (when cuff pressure was measured) varied likewise, with 7% of respondents targeting <20 cm H_2_O, 54% 20–30 cm H_2_O and 34% >30 cm H_2_O. One-fifth to one-fourth of respondents in our survey answered that they did not know the recommended range of cuff pressure for each species they intubated. This reflects the absence of literature data or consensus guidelines on recommended cuff pressure for various species and different makes and sizes of ETT in veterinary medicine. Alternatively, the lack of a significant difference in targeted cuff pressure between species may also reflect a type II error, given the low number of respondents actually measuring cuff pressure. Notably, the >30 cm H_2_O cuff pressure target was selected far more often for horses than for other species. This may indicate that respondents are aware of the higher than 20–30 cm H_2_O cuff pressure needed to achieve a seal with typical equine silicone ETTs, owing to their elastic properties and cuff geometry. In fact, 80 cm H_2_O cuff pressure proved necessary to obtain a tracheal seal sufficient to prevent liquid leakage in horses when a 30 mm silicone ETT was used [9]. Interestingly, if study participants were aware of the discrepancy in cuff pressure needed for silicone vs. PVC ETTs, they apparently did not extend this concept to the silicone ETTs used in farm animals and dogs.

Various techniques can be used and are taught to ascertain a tracheal seal after cuff inflation. The most frequently selected technique in cats, dogs and farm animals was manual syringe inflation of the cuff to minimal occlusive volume (MOV), when gas stopped audibly escaping during a positive pressure breath. In human medicine, this appears to be the safest method to achieve a targeted cuff pressure, other than the use of a manometer [8]. However, in large animals, the MOV technique is very impractical, as a lot of air is required, which could lead to multiple syringe detachments for refilling, which is probably why MOV was not the most frequently selected technique in horses. The fact that the use of a manometer was the least selected option for cuff inflation in horses (5%) in the current survey indicates that cuff pressure measurement is still uncommon in equine practice. This may well be partly due to the absence of guidelines on what exact cuff pressure should be targeted in horses.

Our results revealed very limited use of cuff manometers but common use of MOV to establish a cuff seal in dogs, cats and farm animals. This result is not in line with evidence obtained in humans [27,28,29], as well as in dogs and cats [1,2,30,31] establishing the inferiority of MOV to the use of a manometer in achieving a pre-specified (i.e., target) cuff pressure. Furthermore, when comparing MOV in cats and dogs to the use of commercially available syringe inflation devices, MOV proved inferior [2,32]. Whereas these studies clearly concluded that a cuff manometer should be used to achieve optimal ETT cuff pressure, the use of MOV was three times more common than cuff pressure measurement in dogs and cats in the current study.

Several studies in human medicine have likewise demonstrated pilot balloon palpation to be an unreliable method to assess ETT cuff pressure [8,33,34], proving inferior to other techniques, such as loss of resistance [35], MOV [27] and the use of a manometer [27,36]. In two studies in cats, pilot balloon palpation performed worse than cuff pressure measurement with a manometer in achieving a target cuff pressure [2,31]. Despite these compelling data proving the inferiority of the technique for the purpose of cuff seal assessment, in our survey, pilot balloon palpation was still selected by 13–16% of respondents. It was the third most selected option for cuff seal verification across species and was selected more frequently than the superior technique of cuff pressure measurement.

Diplomates were more likely than other veterinary anaesthesia providers to use ≥3 techniques to establish cuff seal, reflecting that the number of techniques used may be influenced by training level. Whereas the combination of several techniques could prove beneficial in providing an estimate of seal adequacy, particularly in the absence of recommendations for target cuff pressures across species and ETT types, there is no actual evidence to support this notion at the moment. Interestingly, when participants were asked for the reason for their preference of cuff inflation technique, half of them responded that they were taught to do it this way, whereas about one-quarter of respondents indicated their selected techniques were performed by habit. As these answers strongly suggest that veterinary clinical teaching and training have a considerable impact on the technique used to verify tracheal seal after intubation, this provides a direct opportunity for education to help improve practice standards.

Experimental studies have shown that a gradual decrease, as well as fluctuations in cuff pressure, may occur over time in vitro and in intubated humans and animals [37,38,39], presenting a risk for gas and inhalant leakage and/or aspiration. In our survey, when participants were asked if they rechecked the adequacy of the cuff seal, “Yes, sometimes” was the most selected answer option for all species. Hence, it appears that tracheal seal reassessment is not common and certainly not standard practice in veterinary medicine. Years of work experience did not considerably affect standards of rechecking cuff seal adequacy. However, it appeared that training level and practice setting did affect rechecking standards, as the odds of never rechecking the cuff were considerably increased in first-opinion practice in dogs and cats, whereas diplomates were significantly less likely to never recheck the cuff in cats. The odds ratios for diplomates never rechecking the cuff in other species could not be calculated because no diplomate selected this option in any other species. This suggests that advanced anaesthesia training improves anaesthetists’ attention to ETT cuff seal maintenance. With regard to the frequency and timing of cuff seal verification after intubation, respondents most frequently answered that they do this “only on specific indication”, which largely constituted suspicion of anaesthetic gas leakage. Perhaps surprisingly, rechecking cuff seal adequacy within 15 min was more likely to occur in dogs in first-opinion practice, with diplomate status having no effect on the likelihood of rechecking the cuff within 15 min. This may be related to the time taken between induction and theatre transfer (a likely moment for rechecking the cuff after repositioning), as this time frame may be shorter in first-opinion practice than in an university teaching hospital setting. Although guidelines exist for optimal timing and frequency of rechecking the cuff seal, cuff pressure was found to drop significantly in PVC ETTs in Beagle dogs, especially in the first 10 min after the start of anaesthesia [37]. This suggests that it may be sensible to recheck the cuff early and repeatedly. However, every time a cuff inflator is connected to the inflation valve of the ETT pilot balloon, the cuff pressure decreases by an average of 7 cm H_2_O [40]. Whereas continuous cuff pressure measurement [41,42] and adjustment [43] are reliably possible and have been shown to reduce the incidence of ventilator-associated pneumonia in humans [44], this remains an experimental procedure due to the high costs and difficulties associated with practical feasibility.

This study represents a first attempt to evaluate current practice for ETT cuff inflation and seal verification in various veterinary species. Given its survey design and study participant recruitment, there are several limitations that should be noted. In terms of survey design, it should be noted that no formal testing for item reliability nor survey interpretation (legibility testing) was performed, partly due to limited ability to recruit a pilot group of representative respondents. Importantly, we used three different online resources to recruit participants. Two of these (ACVA-L and ECVAA list servers) have an international membership with a special interest and/or advanced training in anaesthesia, whereas the Dutch closed Facebook community (‘Dierenartsengilde’) is aimed at first-opinion veterinarians. This led to the vast majority of respondents being Dutch veterinarians working in first-opinion practice. Therefore, for the results of this survey, we cannot distinguish between the effects of being in first-opinion practice and the effect of being based in the Netherlands on respondent answers. In hindsight, this could have been prevented if we had specifically targeted an international group of anaesthetists working in first-opinion practice, which we might have been able to achieve through the Association of Veterinary Anaesthetists (AVA). Our results are more likely to reflect Dutch first-opinion practice than first-opinion practice in general; further studies should be undertaken targeting an international first-opinion practice population to verify or extend our findings. Based on the Dutch situation, anaesthesia nurses and technicians were treated as one subgroup in the questionnaire, although their responsibilities and legal status can differ between countries. As the subgroup of anaesthesia nurses and technicians made up only 2% of study respondents, this is unlikely to have had an appreciable impact on our results. Furthermore, diplomates were slightly overrepresented in the responses for farm animals and horses; they comprised 54 and 52% of respondents for these species, respectively, versus 31% for both dogs and cats. The percentage of respondents working in first-opinion practice that provided answers for farm animals and horses was very low (2%); hence, answers provided in this survey are not a reliable reflection of first-opinion practice for farm animal and horses. In terms of answer frequencies, statistics on cuff pressure measurement may have suffered from type II error due to the small number of participants who actually measured cuff pressure. As a further limitation, we did not fully stratify for years of work experience; therefore, we cannot rule out effects within or beyond the dichotomized boundaries (e.g., being very recently qualified or having >20 years of experience). Accordingly, the results with regard to the impact of work experience should be interpreted with caution.

Lastly, a question added to the survey about differences in cuff inflation methods for silicone and PVC ETTs specifically would have provided insight with respect to respondent awareness of implications of ETT material for cuff inflation practice.

## 5. Conclusions

Based on the results of this survey, endotracheal tube cuff pressure is not routinely measured in veterinary medicine, and anaesthesia providers seem insufficiently aware of possible differences in endotracheal tube cuff pressure between species and/or make of ETT. Despite literature evidence suggesting cuff pressure measurement by a manometer to be the superior technique for ETT cuff seal verification, this technique is infrequently used. Endotracheal tube cuff pressure is not routinely rechecked in Dutch first-opinion practice, and suspected leakage is the most common reason to recheck the cuff seal. Persistent preference for minimal occlusive volume, pilot balloon palpation and capnogram verification as cuff pressure verification techniques is partly a result of habits but mostly dictated by teaching. Hence, active education and development of evidence-based guidelines could help to improve practice. It should be noted that results from this questionnaire are based on a limited convenience sample with geographical bias and therefore may not be representative of global veterinary practice.

## Figures and Tables

**Table 1:** Response frequency table (percentage of total number of responses) for each species category and statistical significance per questionnaire item between species. Note: For some questionnaire items, study participants could select multiple options; therefore, the total is >100%.

Questionnaire Item	Species			
	Dogs	Cats	Farm Animals	Horses
What material is the endotracheal tube made of?				
Silicone	67.3 ^‡,¶^	45.2 ^†,§,¶^	85.6 ^‡,¶^	91.3 ^†,‡,§^
Rubber	7.7 ^‡^	4.6 ^†^	13.6	12.6
PVC	55.5 ^§,¶^	56.0 ^§,¶^	48.3 ^†,‡,¶^	10.2 ^†,‡,§^
Don’t know	16.2	16.0	0	0.8
Non-selected	2.7 ^§^	0.6	5.1^†^	2.4
Do you reuse the endotracheal tube?				
Yes	76.4 ^§,¶^	75.4 ^§,¶^	89.0 ^†,‡^	96.1 ^†,‡^
No	20.4	22.8 ^§,¶^	3.4 ^‡^	1.6 ^‡^
Other	3.2 ^§,¶^	1.5	3.4 ^†,¶^	0 ^†,§^
Non-selected	0 ^§^	0.3 ^§^	4.2 ^†,‡^	2.4
What method or methods do you use for cuff inflation?				
Syringe filled with air	90.6 ^‡^	87.4 ^†^	90.7	91.3
Syringe filled with fluid	0.9	0.9	0	1.6
Cuff pressure measurement device	30.4	31.7	8.5 ^†,‡^	9.4 ^†,‡^
Other	8.7	0	0	0
Non-selected	0 ^§^	0.9	5.1 ^†^	2.4
How do you measure cuff pressure?				
Manometer	35.9 ^§,¶^	30.1 ^§^	60.0 ^†,‡^	100.0 ^†^
Tru-Cuff ^®^	10.7 ^§,¶^	11.7 ^§,¶^	0 ^†,‡^	0 ^†,‡^
AG Cuffill^®^	35.0 ^‡,§,¶^	41.7 ^†,§,¶^	10.0 ^†,‡^	0 ^†,‡^
PressureEasy ^©^	23.3 ^‡^	18.4 ^†^	0	0
Other	11.7 ^§,¶^	11.7 ^§^	10.0 ^†,‡^	0 ^†^
Non-selected	1.0	0	20.0	0
What is the cuff pressure you use? (if using measurement device)				
Don’t know	7.5	10.7	0	0
<20 cm H2O	3.0	12	12.5	0
20–30 cm H2O	71.6	62.7	62.5	18.2
>30 cm H2O	14.9	14.7	25.0	81.8
Other	3.0	0	0	0
Non-selected	0	0	0	0
What do you think the recommended range of cuff pressure is?				
No answer	23.3 ^§,¶^	24.0 ^§^	16.1 ^†,‡^	18.1 ^†^
Don’t know	15.6 ^§^	15.7 ^§^	5.9 ^†,‡^	3.1
<20 cm H2O	14.2 *	17.5 *	12.7 *	7.9 *
20–30 cm H2O	31.6 ^§^	28.6	36.4 ^†^	38.6
>30 cm H2O	10.3 ^§,¶^	7.7 ^§,¶^	23.7 ^†,‡^	26.0 ^†,‡^
Other	5.0	5.5	5.1	6.3
Non-selected	0	0	0	0
What techniques do you use to establish a good seal of the cuff?				
Minimal Occlusive Volume	35.8 ^§,¶^	38.0 ^§,¶^	27.5 ^†,‡,¶^	17.6 ^†,‡,§^
Absence smell of inhalational anaesthetic	6.2 ^§,¶^	7.4 ^§,¶^	11.7 ^†,‡,¶^	15.0 ^†,‡,§^
Palpation pilot balloon	14.5	14.1	16.1	13.4
Loss of resistance	1.9 ^§,¶^	0 ^†^	0 ^†^	0 ^†^
Syringe pressure	4.5 ^¶^	3.8 ^¶^	5.1	8.2 ^†,‡^
Filling of the ventilator bellows	3.9 ^§,¶^	3.7 ^§,¶^	12.4 ^†,‡,¶^	17.6 ^†,‡,§^
Verification normal capnogram waveform	20.7 ^‡^	19.2 ^†^	20.9	19.9
Measurement cuff pressure	11.5 ^§,¶^	12.9 ^§,¶^	4.8 ^†,‡^	4.6 ^†,‡^
Auscultation trachea	1.1	1.0	1.5 ^¶^	3.6 ^§^
Other	4.4	4.0	3.4	5.5
Non-selected	0.9 ^‡,§^	1.8 ^†^	7.6 ^†^	7.1
Why do you use this technique?				
No specific preference	7.2	8.5	13.1	10.3
By habit	25.9	26.1	26.3	26.6
I was taught to do it this way	47.7 ^‡^	47.1 ^†^	46.7 ^¶^	54.0 ^§^
Literature	13.3 ^§,¶^	13.3 ^§,¶^	7.9 ^†,‡^	5.5 ^†,‡^
Other	5.9	5.0	5.9	3.6
Non-selected	0	0	0	0
Do you re-check the cuff?				
No, never	8.4	9.3	1.6	1.5
Yes, sometimes	48.9	49.9	46.7	53.8
Yes, frequently	19.0 ^‡,¶^	15.5 ^†,¶^	21.3	11.4 ^†,‡^
Yes, always	8.3	8.4	13.2	12.1
Only with specific indications	15.3 ^§,¶^	16.9 ^¶^	17.2 ^†^	21.2 ^†,‡^
Non-selected	0	0	0	0
When do you re-check the cuff after endotracheal intubation?				
≤15 min	11.7 ^¶^	14.1	12.7	16.5 ^†^
>15 to ≤30 min	24.4 ^¶^	25.1 ^¶^	28.2 ^¶^	14.9 ^†,‡,§^
>30 min	9.1	8.2	10.9	12.4
Only with specific indications	46.9	46.4	42.7	48.8
Other	7.8 ^§^	6.2	5.4^†^	7.4
Non-selected	0	0	0	0
What techniques do you use to establish a good seal of the cuff?				
Minimal Occlusive Volume	57.3 ^§,¶^	94.3 ^§,¶^	53.4 ^†,‡,¶^	36.0 ^†,‡,§^
Absence smell of inhalational anaesthetic	30.1 ^§,¶^	29.7 ^§,¶^	38.8 ^†,‡^	40.0 ^†,‡^
Palpation pilot balloon	33.3	33.1	36.2	31.2
Loss of resistance	3.6 ^§,¶^	2.0 ^§,¶^	14.7 ^†,‡^	18.4 ^†,‡^
Syringe pressure	6.8 ^¶^	4.8	10.3	11.2 ^†^
Filling of the ventilator bellows	20.1 *	16.4 *	42.2 *	52.8 *
Verification normal capnogram waveform	52.4 ^‡^	48.8 ^†^	50.9	56.0
Measurement cuff pressure	20.1 ^‡,§,¶^	26.6 ^†,§¶^	12.1 ^†,‡^	8.8 ^†,‡^
Auscultation trachea	2.3	2.7	3.4	5.6
Other	5.5	4.4	2.6 ^¶^	5.6 ^§^
Non-selected	9.7	10.5	6.8	4.7

PVC = polyvinyl chloride; * significant difference between all species within the same questionnaire item (*p* < 0.05); ^†^ significant difference from dogs within the same questionnaire item (*p* < 0.05); ^‡^ significant difference from cats within the same questionnaire item (*p* < 0.05); ^§^ significant difference from farm animals within the same questionnaire item (*p* < 0.05); ^¶^ significant difference from horses within the same questionnaire item (*p* < 0.05).

**Table 2:** Odds ratio for frequency of responses for diplomates, first-opinion practitioners and respondents more than 10 years of experience for selected questionnaire items.

	Diplomate			First-Opinion Practice			>10 Years of Experience		
		95% Confidence Interval			95% Confidence Interval			95% Confidence Interval	
	Odds Ratio	Lower	Upper	Odds Ratio	Lower	Upper	Odds Ratio	Lower	Upper
Species									
Dogs	0.897	0.220	3.657	2.648	0.542	12.933	0.364	0.075	1.780
Cats	1.031	0.411	2.583	0.959	0.409	2.252	1.460	0.626	3.406
Farm Animals	5.010	3.072	8.170	0.010	0.002	0.041	1.463	0.929	2.304
Horses	4.611	2.842	7.481	0.008	0.002	0.034	1.364	0.875	2.128
Reuse of endotracheal tubes									
Dogs	1.221	0.745	2.001	0.980	0.614	1.654	1.316	0.773	2.241
Cats	1.785	1.090	2.924	0.852	0.530	1.371	1.280	0.759	2.157
Farm Animals	1.059	0.270	4.159	*	*	*	0.588	0.059	5.850
Horses	0.917	0.279	3.011	*	*	*	1.027	0.990	1.065
Measuring cuff pressure									
Dogs	1.221	0.745	2.001	0.980	0.614	1.654	1.320	0.823	2.117
Cats	1.785	1.090	2.924	0.852	0.530	1.371	1.712	1.054	2.783
Farm Animals	1.059	0.270	4.159	*	*	*	0.882	0.235	3.316
Horses	0.917	0.279	3.011	*	*	*	0.868	0.259	2.903
≥3 techniques to verify cuff seal									
All species	2.075	1.307	3.294	0.453	0.288	0.710	1.152	0.746	1.778
Never rechecking the cuff									
Dogs	**	**	**	10.238	3.486	30.063	0.303	0.134	0.683
Cats	0.062	0.008	0.463	8.854	3.313	23.662	0.547	0.262	1.142
Farm Animals	**	**	**	*	*	*	0.955	0.895	1.018
Horses	**	**	**	*	*	*	0.623	0.038	10.202

A table presenting all odds ratios calculated from the survey data can be found in Appendix B. * It is not possible to calculate a reliable odds ratio for farm animals and horses, owing to a very limited number of non-diplomate respondents in these species categories; ** it is not possible to calculate an odds ratio, as no diplomates answered that they never rechecked the cuff.

## Data Availability

Not applicable.

## Appendix A. Questionnaire

Demographic

1. What is your gender?
  - Female
  - Male
  - Non-binary/third gender
  - Prefer not to say

2. In which country do you currently work?
3. What is your job title?
  - Veterinarian
  - Anaesthesiology nurse/technician
  - Anaesthesiology Intern
  - Anaesthesiology Resident
  - ACVAA diplomate
  - ECVAA diplomate
  - ECC nurse/technician
  - ECC veterinarian
  - ACVECC diplomate
  - ECVECC diplomate
  - Other (please specify)

4. What is your place of work?
  - First opinion practice
  - Referral practice
  - University hospital
  - Other (please specify)

5. How many years of veterinary experience do you have?
  - ≤2 years
  - >2 years to ≤5 years
  - >5 years to ≤10 years
  - >10 years

6. In which animal species do you perform endotracheal intubation? (multiple options possible)
  - Dogs
  - Cats
  - Farm Animals
  - Horses

Cuff related questions per species

1. What material is the endotracheal tube for [species] made of? (multiple options possible)
  - Silicone
  - Rubber
  - PVC
  - Don’t know

2. Do you reuse the endotracheal tube for [species]?
  - Yes
  - No
  - Other (please specify)

3. What method or methods do you use for cuff inflation in [species]? (multiple options possible)
  - Syringe filled with air
  - Syringe filled with fluid
  - Cuff pressure measurement device
  - Other (please specify)

4. * How do you measure cuff pressure in [species]? (multiple options possible)
  - Manometer
  - Tru-Cuff ^®^
  - AG Cuffill^®^
  - PressureEasy ^©^
  - Other (please specify)

5. What techniques do you use to establish a good seal of the cuff in [species]? (multiple options possible)
  - Minimal occlusive volume (=inflating cuff until no audible leak is present)
  - Absence of smell (=inflation cuff until absence of the smell of inhalational anaesthetic)
  - Palpation pilot balloon
  - Loss of resistance (=hyperinflation cuff and then allowing passive release of all excess air until the plunger of the syringe stops moving)
  - Syringe pressure (=cuffing to a cuff pressure by feeling the pressure in the syringe)
  - Filling of the ventilator bellows
  - Verification of normal capnogram waveform
  - Measurement of cuff pressure
  - Auscultation of the trachea
  - Other (please specify)

6. Why do you use this technique in [species]? (multiple options possible)
  - No specific preference
  - By habit
  - I was taught to do it this way
  - I have read in the literature that this was the recommended method
  - Other (please specify)

7. * What is the cuff pressure you use in [species]? (please include an unit, e.g., kPa, mmHg or cmH_2_O)
8. Do you re-check the cuff in [species] (at a later time point)? (multiple options possible)
  - No, never
  - Yes, sometimes
  - Yes, frequently
  - Yes, always
  - Only with specific indications (please specify)

9. ** When do you re-check the cuff in [species] after endotracheal intubation?
  - ≤15 min
  - >15 to ≤30 min
  - >30 min
  - Only with specific indications (please specify)
  - Other (please specify)

10. ** How do you re-check the cuff in [species]?
  - Minimal occlusive volume (=inflating cuff until no audible leak is present)
  - Absence of smell (=inflation cuff until absence of the smell of inhalational anaesthetic)
  - Palpation pilot balloon
  - Loss of resistance (=hyperinflation cuff and then allowing passive release of all excess air until the plunger of the syringe stops moving)
  - Syringe pressure (=cuffing to a cuff pressure by feeling the pressure in the syringe)
  - Filling of the ventilator bellows
  - Verification of normal capnogram waveform
  - Measurement of cuff pressure
  - Auscultation of the trachea
  - Other (please specify)

11. What do you think the recommended range of cuff pressure is in [species]? (please include an unit, e.g., kPa, mmHg or cmH_2_O)
12. Please place the following items associated with endotracheal tube cuffing in order of highest (1) to lowest (4) perceived importance in [species].
  1. Decrease the risk of aspiration
  2. Avoiding contamination of working environment with anaesthetic gas
  3. Ability to provide mechanical ventilation
  4. Avoiding tracheal mucosal damage

* questions was only available when in question 3 “cuff pressure measurement device” was selected.

** question was only available when in question 8 “no, never” was not selected.

## Appendix B. Odds Ratio for Frequency of Response for Diplomates, First Opinion Practitioners and More than 10 Years of Experience for Selected Questionnaire Items

**Table:**

	Diplomate			First-Opinion Practice			>10 Years’ Experience		
		95% Confidence Interval			95% Confidence Interval			95% Confidence Interval	
	Odds Ratio	Lower	Upper	Odds Ratio	Lower	Upper	Odds Ratio	Lower	Upper
Species									
Dogs	0.897	0.220	3.657	2.648	0.542	12.933	0.364	0.075	1.780
Cats	1.031	0.411	2.583	0.959	0.409	2.252	1.460	0.626	3.406
Farm Animals	5.010	3.072	8.170	0.010	0.002	0.041	1.463	0.929	2.304
Horses	4.611	2.842	7.481	0.008	0.002	0.034	1.364	0.875	2.128
Reuse of endotracheal tubes									
Dogs	1.221	0.745	2.001	0.980	0.614	1.654	1.316	0.773	2.241
Cats	1.785	1.090	2.924	0.852	0.530	1.371	1.280	0.759	2.157
Farm Animals	1.059	0.270	4.159	*			0.588	0.059	5.850
Horses	0.917	0.279	3.011	*			1.027	0.990	1.065
Measuring cuff pressure									
Dogs	1.221	0.745	2.001	0.980	0.614	1.654	1.320	0.823	2.117
Cats	1.785	1.090	2.924	0.852	0.530	1.371	1.712	1.054	2.783
Farm Animals	1.059	0.270	4.159	*			0.882	0.235	3.316
Horses	0.917	0.279	3.011	*			0.868	0.259	2.903
≥ 3 techniques to verify cuff seal									
All species	2.075	1.307	3.294	0.453	0.288	0.710	1.152	0.746	1.778
Never rechecking the cuff									
Dogs	**			10.238	3.486	30.063	0.303	0.134	0.683
Cats	0.062	0.008	0.463	8.854	3.313	23.662	0.547	0.262	1.142
Farm Animals	**			*			0.955	0.895	1.018
Horses	**			*			0.623	0.038	10.202
Yes, sometimes									
Dogs	1.104	0.696	1.752	0.936	0.609	1.439	1.096	0.714	1.684
Cats	1.250	0.779	2.005	0.792	0.510	1.231	1.114	0.717	1.729
Farm Animals	1.331	0.643	2.753	*			0.499	0.234	1.065
Horses	0.891	0.442	1.798	*			0.611	0.294	1.270
Yes, frequently									
Dogs	2.063	1.193	3.568	0.334	0.182	0.614	1.570	0.904	2.725
Cats	1.429	0.774	2.639	0.478	0.251	0.910	1.168	0.641	2.128
Farm Animals	0.804	0.336	1.922	*			3.091	1.071	8.915
Horses	1.447	0.483	4.337	*			1.847	0.553	6.164
Yes, always									
Dogs	1.126	0.508	2.498	0.372	0.155	0.893	2.308	0.997	5.344
Cats	1.186	0.530	2.651	0.324	0.128	0.819	2.084	0.894	4.857
Farm Animals	0.821	0.286	2.359	*			0.990	0.333	2.940
Horses	0.685	0.239	1.970	*			1.445	0.470	4.443
Only with specific indications									
Dogs	0.646	0.331	1.262	2.727	1.499	4.964	0.656	0.368	1.171
Cats	0.998	0.540	1.843	1.577	0.892	2.789	0.701	0.396	1.238
Farm Animals	4.521	1.418	14.418	*			2.982	0.933	9.531
Horses	2.336	0.963	5.664	*			2.815	1.050	7.549
Recheck cuff <15 min									
Dogs	0.529	0.232	1.207	2.138	1.059	4.314	0.881	0.437	1.774
Cats	0.573	0.269	1.224	1.693	0.871	3.291	1.006	0.514	1.966
Farm Animals	0.836	0.320	2.184	*			0.667	0.213	2.085
Horses	0.684	0.222	2.105	5.263	0.315	87.848	0.707	0.268	1.863

* It is not possible to calculate a reliable odds ratio for farm animals and horses due to a very limited number of non-diplomate respondents in these species categories; ** it is not possible to calculate an odds ratio, as no diplomates answered that they never rechecked the cuff.

## Appendix C. Demographics of Study Participants

**Table A1:** Gender.

	Frequency	Percentage (%)
Female	259	74.4
Male	83	23.9
Non-binary/third gender	0	0
Prefer not to say	6	1.7

**Table A2:** Country.

	Frequency	Percentage (%)
Australia	14	4.0
Austria	1	0.3
Belgium	10	2.9
Canada	10	2.9
Colombia	1	0.3
Finland	1	0.3
France	3	0.9
Germany	6	1.7
Greece	1	0.3
Hong Kong (S.A.R.)	1	0.3
Ireland	3	0.9
Israel	1	0.3
Italy	11	3.2
Netherlands	163	46.8
New Zealand	1	0.3
Norway	2	0.6
South Africa	2	0.6
South Korea	1	0.3
Spain	6	1.7
Sweden	3	0.9
Switzerland	9	2.6
United Kingdom of Great Britain and Northern Ireland	42	12.1
United States of America	56	16.1

**Table A3:** Job title.

	Frequency	Percentage (%)
Job title		
Veterinarian	182	52.3
Anaesthesiology nurse/technician	7	2.0
Anaesthesiology intern	1	0.3
Anaesthesiology resident	34	9.8
ACVAA diplomate	48	13.8
ECVAA diplomate	61	17.5
ECC nurse/technician	0	0
ECC veterinarian	1	0.3
ACVECC diplomate	0	0
ECVECC diplomate	0	0
Other	14	4.0
Job title (other).		
Specialist small animal surgery	1	7.1
Anaesthesiology professor	2	14.2
PhD anaesthesia	1	7.1
Residency trained anaesthesiologist	10	71.4

**Table A4:** Place of work.

	Frequency	Percentage (%)
Place of work		
First-opinion practice	151	43.4
Referral practice	75	21.6
University hospital	110	31.6
Other	12	3.4
Place of work (other)		
University and referral practice	2	16.7
University and first-opinion practice	1	8.3
First-opinion and referral practice	1	8.3
Consultant	2	16.7
Executive	1	8.3
Research organisation	2	16.7
Preclinical industry	1	8.3
Retired	1	8.3

**Table A5:** Years of experience.

	Frequency	Percentage (%)
≤2 years	38	10.9
>2 years to ≤5 years	38	10.9
>5 years to ≤10 years	75	21.6
>10 years	197	56.6

**Table A6:** Animal intubations performed.

	Frequency	Percentage per Species	Percentage of Total
Dogs	339	97.4	37.3
Cats	325	93.4	35.8
Farm animals	118	33.9	13.0
Horses	127	36.5	14.0
All four species	111		31.9

**Table A7:** Frequency and percentage of diplomates per species.

	Frequency	Percentage per Species	Frequency (Total)
Dogs	105	31.0	339
Cats	101	31.1	325
Farm animals	64	54.2	118
Horses	66	52.0	127

**Table A8:** Frequency and percentage of diplomates per country.

	Frequency	Percentage (%)
Australia	11	10.2
Austria	0	0
Belgium	2	1.9
Canada	6	5.6
Colombia	0	0
Finland	1	0.9
France	1	0.9
Germany	3	2.8
Greece	1	0.9
Hong Kong (S.A.R.)	1	0.9
Ireland	3	2.8
Israel	1	0.9
Italy	11	10.2
Netherlands	1	0.9
New Zealand	0	0
Norway	1	0.9
South Africa	1	0.9
South Korea	0	0
Spain	2	1.9
Sweden	2	1.9
Switzerland	6	5.6
United Kingdom of Great Britain and Northern Ireland	24	22.2
United States of America	37	34.3
Total	108	

**Table A9:** Frequency and percentage of first-opinion practice per species.

	Frequency	Percentage per Species	Frequency (Total)
Dogs	146	43.1	339
Cats	138	42.5	325
Farm animals	2	1.7	118
Horses	2	1.6	127

**Table A10:** Frequency and percentage of job title of Dutch compared to non-Dutch respondents.

	Frequency Dutch	Percentage Dutch	Frequency Non-Dutch	Percentage Non-Dutch
Veterinarian	157	45.1	25	7.2
Anaesthesiology nurse/technician	0	0	7	2.0
Anaesthesiology intern	1	0.3	0	0
Anaesthesiology resident	2	0.6	32	9.2
ACVAA diplomate	0	0	48	13.8
ECVAA diplomate	1	0.3	60	17.2
ECC nurse/technician	0	0	0	0
ECC veterinarian	1	0.3	0	0
ACVECC diplomate	0	0	0	0
ECVECC diplomate	0	0	0	0
Other	1	0.3	13	3.7
Total	163	46.8	185	53.1

**Table A11:** Frequency and percentage of Dutch respondents working in first-opinion practice compared to total respondents working in first-opinion practice.

	Frequency	Percentage
Dutch respondents	163	46.8
Total respondents	348	100
Dutch respondents working in first-opinion practice	143	94.7
Total respondents working in first-opinion practice	151	100
